# Supramolecular Coordination Assemblies Constructed From Multifunctional Azole-Containing Carboxylic Acids

**DOI:** 10.3390/molecules15053478

**Published:** 2010-05-12

**Authors:** Yuheng Deng, Hao Liu, Bo Yu, Min Yao

**Affiliations:** Department of Chemistry, Capital Normal University, No. 105, Xisanhuan Beilu, Haidian District, Beijing, 100048, China

**Keywords:** multifunctional ligands, azole-containing carboxylic acids, coordination polymers, topology

## Abstract

This paper provides a brief review of recent progress in the field of metal coordination polymers assembled from azole-containing carboxylic acids and gives a diagrammatic summary of the diversity of topological structures in the resulting infinite metal-organic coordination networks (MOCNs). Azole-containing carboxylic acids are a favorable kind of multifunctional ligand to construct various metal complexes with isolated complexes and one, two and three dimensional structures, whose isolated complexes are not the focus of this review. An insight into the topology patterns of the infinite coordination polymers is provided. Analyzed topologies are compared with documented topologies and catalogued by the nature of nodes and connectivity pattern. New topologies which are not available from current topology databases are described and demonstrated graphically.

## 1. Introduction 

The assembly, structure and potential applications of metal-organic coordination polymers, especially the Metal-Organic Frameworks (MOFs), as functional materials have attracted extensive attention from researchers worldwide because of their intriguing complicated compositions, versatile framework topologies and interesting properties in gas sorption, optics, magnetism and as supporting carriers, *etc.* [1,2,3,4,5,6,7]. At present, the rational synthetic strategy in this field usually involves the use of multifunctional ligands with multiple active coordination sites to prepare the target compound [8,9,10,11,12]. Polydentate ligands can act as either bridging or chelating ligands to link metal ions together, resulting in the desired networks in the final metal organic coordination polymers [13,14,15,16]. 

According to a statistical analysis of the literature over the past decade, multifunctional carboxylate ligands with nitrogen-bearing heterocycles have been used expansively in the synthetic strategies to develop multidimensional (one, two and three dimensional) framework structures. For example, pyridinecarboxylic acid and its analogues with active oxygen and nitrogen sites on the both ends have been successfully applied to synthesize coordination polymers [17,18,19,20,21,22,23,24,25,26,27,28]. Compared to the above rigid ligands, the relatively flexible and peculiar carboxylic acids derived from the diazole, triazole and tetrazole moieties have come to be regarded as all-purpose ligands in recent years that can potentially coordinate metal ions in various ways, due to their complicated coordination modes and different performance of the N and O ends. The resulting product generally has various structures with distinct topology. A longer and flexible spacer between the N and O end may even result in more complicated topological forms with multiple interpenetrations. These interpenetrating networks of coordination polymers are also an interesting focus of attention currently [29]. For example, the multifunctional ligand terazole-1-acetic acid (Htza) has been used successfully in the synthesis of a series of coordination polymers [30,31,32,33,34,35,36,37,38,39,40,41,42,43,44,45,46,47,48,49,50,51] for its variety of coordination styles on the tetrazole and carboxylate group ends. Recently, we and Yu *et al*. independently and simultaneously synthesized a series of Cu^II^ compounds assembled with Htza and published the analysis of their magnetic properties [51,40].

In this paper, our discussion will focus on the topological structure of the MOCNs constructed by the multifunctional carboxylate ligands containing five-membered N-heterocyclic rings (azoles). The azoles in question include 1,2-diazoles (pyrazole), 1,3-diazoles (imidazole), 1,2,3,-triazoles, 1,2,4-triazoles and tetrazoles. Over the about past decade, a significant number of metal complexes formed by the title ligands with one-, two-, or three-dimensional framework structures have been reported. Quite many among these are zero-dimensional finite structures, which are not our interest in this work. Most of the zero-dimensional compounds are binuclear. The highest-nuclearity of the reported oligomeric complexes up to now is octanuclear, with cubic structure [52,53,54,55]. A summary and discussion for the variety of the topology patterns of the MOCNs assembled by the azole-containing carboxylic acid is contributed to this paper.

**Scheme 1:**
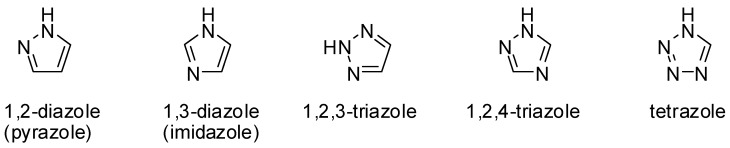
The types of azole considered.

## 2. Methodology

Structural data was retrieved from Cambridge Structure Database [56,57,58] (CSD) up to May 2009. Only azole-containing carboxylic acid ligands in which both nitrogen atoms in the azole ring and oxygen atom in the carboxylic acid group are coordinated to metal atoms are selected to perform further topological analysis. Nearly three hundred corresponding structures that were published ranging from 1967 to 2009 are listed, in which 81(28%) are zero-dimensional complexes, 71(24%) are one-dimensional structures, 61(21%) are two-dimensional layers and 77(27%) are three-dimensional nets.

In general, MOCNs are constituted by two main parts: the organic linkers and the metal ions. Both metal atoms and ligands are considered as nodes. Coordination bonds are considered as links between nodes. Analysis of topology is performed by using the compiled topology and tiling analytical software TOPOS [59]. As the first step, determination of bonding interactions is calculated by the AutoCN subprogram using the Sectors algorithm in which an improved method of intersecting spheres designed by Peresypkina and Blatov [60] for organic and metal-organic compounds is used. In this method, which is call *method of spherical sectors*, a sphere of *R_sd_* radius is replaced with a set of spherical sectors corresponding to interatomic contacts. The radius (*r_sec_*) of the *i*th sector is determined by the formula $r_{\sec}={\left(\frac{3v_{i}}{\Omega_{i}}\right)}^{\frac{1}{3}}$, where *V_i_* and Ω*_i_* are volume and solid angle of a pyramid with basal Voronoi-Dirichlet polyhedron (VDP) face corresponding to interatomic contacts and with the VDP atom in the vertex. 

Then both metal atoms and ligands are considered as nodes and the position of nodes that representing clusters or ligands are positioned at the centroids of the group. Coordination bonds are considered as links between nodes. All 0-connected (isolated), 1-connected (dangling) and 2-connected (bridging) nodes are removed to simplify the topology to a maximum extent. 

Determination of topology and taxonomy of the simplified nets are analyzed by a subprogram named ADS and searched in the TTD (TOPOS Topological Database) collections for same topological descriptors. The topology of the three-dimensional nets are described by Point Symbol [61], also known as Schläfli Symbol [62], which lists the numbers and sizes of circuits (closed chains of connected atoms) starting from any non-equivalent atom in the net. Instead of Point Symbol, Vertex Symbol, which enumerates the size of faces around each kind of vertex in cyclic order, is adopted customarily to represent topology of two-dimensional sheets. A few exceptions of nonplanar two-dimensional structures, which are two-dimensional but can’t be realized in a plane without intersection of edges, are described by Point Symbol. Known topologies are characterized by RCSR [63] lower-case three-letter symbols, see http://rcsr.anu.edu.au/ for details.

## 3. One-Dimensional Coordination Chains 

### 3.1. Chain

**Figure 1:**
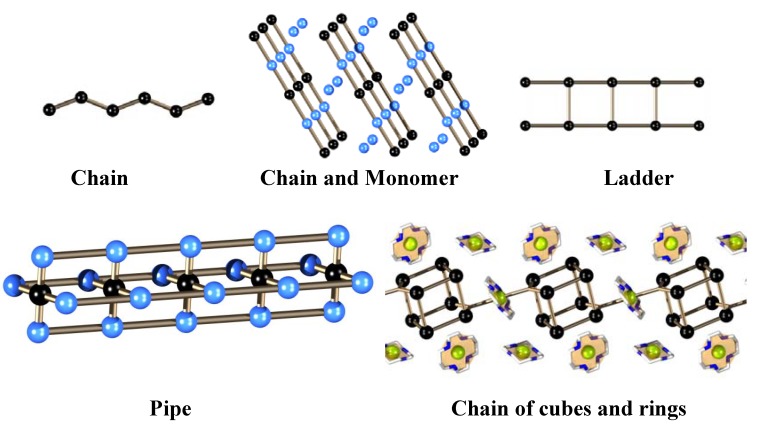
Topology of one-dimensional coordination polymers.

Single chain is the most preferred one-dimensional structure which can be catalogued into several groups (see **Chain** in Figure 1): with the title molecules bridging a zigzag chain of metal atoms, one bulky ligand or several small solvent ligands seal the opening of metal atoms. One title ligand links three metal atoms and one metal atom links three ligands, step by step a one-dimensional chain is formed.

The CSD Refcodes presenting in each type of topology are listed below:
CICZUJ[64]OGALEO[71]FEGGAA[78]MEFZUS[87]VIKCOI[97]CEYLOI[104]PIFBAI[65]OGALIS[71]FOHFUE[79]MENGAO[88]VIQYEA[98]ECULUK[105]PODZOY[66]OKEHIV[72]GOYSES[80]NENDAL[89]VIQYIE[98]OKEHIV01[67]AVUPUC[67]OKEHOB[72]GOYSIW[80]NIQGUQ[90]WUPHEU[99]RAJNOG[106]AVUQOX[67]VOBKUT[73]HIHJIS[81]PAJJOA[91]XIBPAA[100]KOBGUE[40]DOGMAO[68]BIPJEQ[74]IDIXOJ[82]PAJJOA01[92]XIKWAQ[101]
DOGMES[68]DATMUH[75]IYASEG[83]PEXSIV[93]YIFQUZ[102]
HUXTUP[69]DATNAO[75]LAJZIG[84]PEXSIV01[94]TIWRUN[103]
NIQWUG[70]DOGZAB[76]LAQPAV[85]QANDUF[95]KEXWIU[37]
OGAKUD[71]EDURUR[77]LASSEE[86]SENJEB[96]PEXVEU[37]


### 3.2. Chain and monomer

As a rare case, CEYLEY is an interesting structure of mononuclear complexes of two title ligands and a copper atom are linked by six coordinated tin atoms into a single chain coordinated topology while between chains separated mononuclear complexes bind them into a two-dimensional **sql** layer by hydrogen bonds (see **Chain and Monomer** in Figure 1). π-π stacking of diazole rings exists in the piling of layers.
CEYLEY[104]

### 3.3. Chains of cubes and rings

Octanuclear oligomer of cobalt atoms and titled ligands are linked by nickel atoms with tetradentate ring-style ligands into an infinite chain structure that forms a very interesting heterometallic structure (see **Chains of cubes and rings** in Figure 1).
WIBNOL[107]

### 3.4. Ladder

In the ladder structure (see **Ladder** in Figure 1), metal atoms are 3-connected nodes, where the title ligands are vertical linkers, and small molecules (such as water, oxalic acid) of metal-metal bonds are horizontal linkers. In the DILGIP, POHSUB and VODCEX structures, metal atoms and title ligands serve as two counterpart 3-connected nodes. The vertical linker of PEFVIF is a Second Building Unit (SBU) constituted by a dinuclear complex.
ABAYEI[108]XENCEZ[109]DILGIP[111]KEPYIO[113]ROMRUH[115]LIWLUZ[36]AVUQAJ[67]XOKRIZ[110]HOSTEO[112]POHSUB[114]PEFVIF[116]VODCEX[43]

### 3.5. Pipe

Four single chains are linked into one bamboo-like pipe (see **Pipe** in Figure 1) with the title ligandd as chain linkers and joints of the hollows.
YIFSIP[102]

All these topologies are shown in Figure 1. 

## 4. Two-Dimensional Coordination Layers 

### 4.1. sql

**Figure 2:**
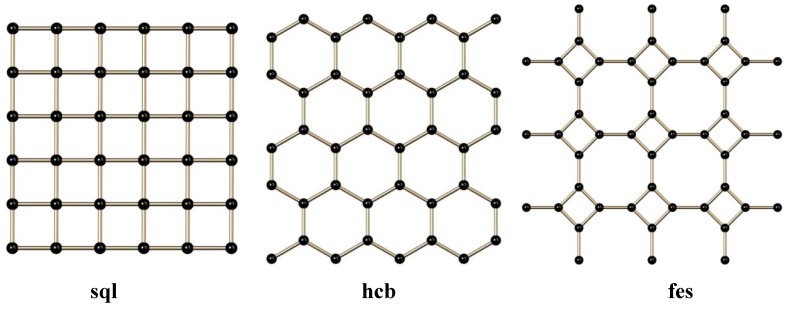
Topology of the uninodal two-dimensional coordination polymers.

The uninodal topology **sql** is a most common one, whose shape is a square grid sheet and has a vertex symbol of {4^4^}(see **sql** in Figure 2). Most of the structures of **sql** topology represent 4-connected metal atoms as vertexes and 2-connected ligands as edges. Both metal atoms and ligands serve as 4-connects nodes in OFITAZ and PEZROC. The structures LIQVEN, YASSEQ, ODIVIH and ODIVON show interweaved **sql** topology.
TIGCAO[117]JEXSIP[120]LIQVEN[122]SONJUA[126]POLDIE[129]MISHAY[50]EVONOS[118]KEPYOU[121]LIWKIM[123]YASSEQ[127]KOBGOY[40]PEZROC[38]HOGDEN[114]KEPYOU01[68]OFITAZ[124]ODIVIH[128]KOBGOY01[42]XOHPAM[48]JEDYEX[119]LAQNUN[85]OFITAZ01[125]ODIVON[128]LIWLOT[36]XOHPEQ[48]

### 4.2. hcb

**hcb** is also a very common uninodal planar topology with a vertex symbol of {6^3^} and a honeycomb-like shape (see **hcb** in Figure 2). BOKXUV, FIBJEG, KEKWIH, TIVZII, TIWBAD, VIQZAX, WOFVET and EHAGAW have metal atoms as all 3-connected nodes and ligands as linkers. FENSUN use both metal atoms and the title ligands as 3-connected nodes. NETXIU and NOFGAR use the title ligands as 3-connected nodes and metal atoms with terminal water as linkers.
BOKXUV[130]FIBJEG[133]TIVZOO[132]WOFVET[94]NOFGAR[136]FENSUN[131]KEKWIH[134]TIVZUU[132]VIQZAX[98]NOFGEV[136]FENSUN03[132]TIVZII[132]TIWBAD[132]NETXIU[135]EHAGAW[137]

### 4.3. fes

**fes** is a {4.8^2^} topology planar structure with one type of 3-connected nodes. All **fes** structure is formed by both the title ligands and metal atoms serve as 3-connected nodes and one or two terminal waters on the metal by the side of a two-dimensional sheet (see **fes** in Figure 2).
FENSUN01[138]OFIVIJ[125]TIWBEH[132]YELYIY01[140]FENSUN02[92]QEXPAL[139]YELYIY[92]LIMNOL[35]

### 4.4. kgd

**Figure 3:**
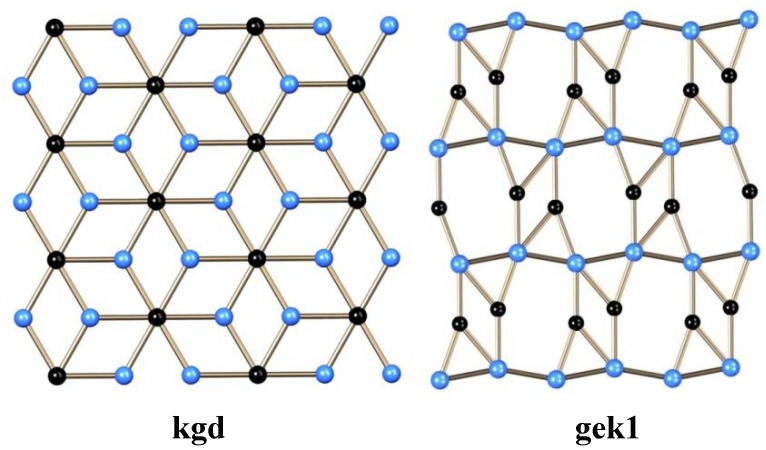
Topology of binodal two-dimensional coordination polymers.

Topology **kgd** has a vertex symbol of {4^3^}_2_{4^6^}, with two type of nodes, 3-connected and 6-connected nodes. All structures with **kgd** topology in this research have the same ligands and configuration in which the ligand serves as a 3-connected node and metal atoms as a 6-connected node (see **kgd** in Figure 3).
JEXSAH[120]JEXSEL[120]SEYVEY[141]

### 4.5. gek1

**gek1** is a binodal two-dimensional topology in *personal.ttd* database in the TTD collection with the vertex symbol of {3.4.6}{3.4.6.3.6} (see **gekl** in Figure 3). TIKWUG is basically a title ligand bridged metal-acetic acid chain which is constructed of 5-connected cadmium atoms and 3-connected titled ligands and 2-connected acetic acids.TIKWUG[142]

### 4.6. New two-dimensional topologies

Six new topologies which are not present in the TTD collection are listed below (Shown in Table 1). 

## 5. Three-Dimensional Coordination Frameworks 

### 5.1. dia

**Figure 4:**
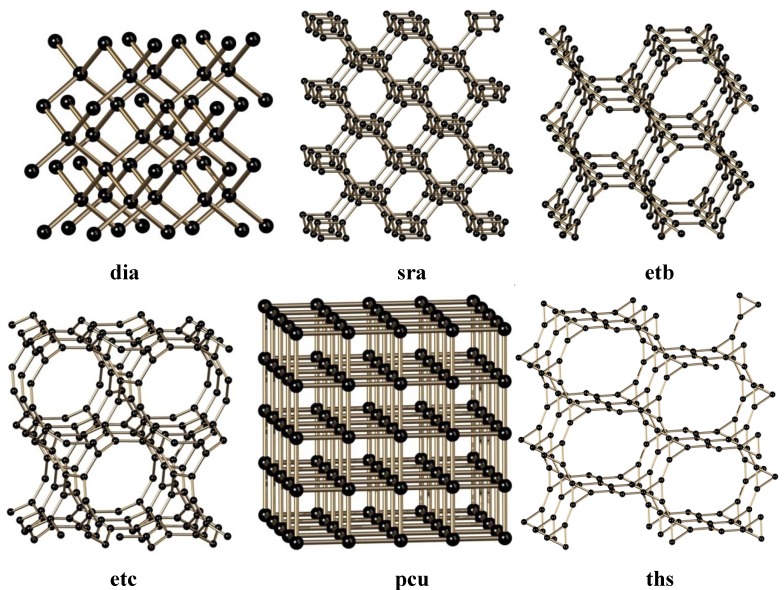
Topology of the uninodal three-dimensional networks.

**dia** is a most common 3-D uninodal topology with the point symbol of {6^6^} (see **dia** in Figure 4). It contains one kind of 4-c node. All target structures with **dia** topology have an interpenetration style, which is caused by the large porous structure of a single **dia** framework. Rather than the three penetrated framework in AGOMOZ and SEYVIC, the four penetrated framework in LUMDEC, METYIU and NEHZIK, is preferred, which may stem from the presence of a slimmer ligand. In all the six **dia** structures (Figure 4), metal atoms are the 4-connected nodes and 2-connected ligands serve as edges.
AGOMOZ[149]SEYVIC[150]LUMDEC[150]LUMDIG[150]METYIU[151]NEHZIK[152]

### 5.2. sra

**sra** is a uninodal 4-connected topology with the point symbol of {4^2^.6^3^.8} (see **sra** in Figure 4). Four out of five of the **sra** structures consist of tetrazole-containing ligands. In all the result, both metal atoms and ligands serve as 4-connected nodes and few has coordinative water on metal atoms.
RAPBEP[153]GAMFEG[154]INOXUE[30]INOYAL[30]KOCWAB[41]QEYXAU[155]

### 5.3. etb

**etb** is a uninodal topology with one kind of 3-connected node and its point symbol is {8^3^} and vertex symbol is [8.8.8(2)] (see **etb** in Figure 4). Both of the cadmium atoms and titled ligands in VERQOZ are 3-connected vertexes with terminal pyridines on the metal atoms.
VERQOZ[156]

### 5.4. etc

**etc** is also a uninodal topology with one kind of 3-connected node and its point symbol is {8^3^} and vertex symbol is [8.8.8(2)] (see **etc** in Figure 4), which are same the **etb**, but they represent different topologies. Both of the manganese atoms and title ligands in WOMFIO are 3-connected vertexes leading to three-dimensional porous structure in which small solvent molecules are contained.
WOMFIO[157]

### 5.5. pcu

**pcu** (primitive cubic), another common topology, is a 6-connected uninodal net with the point symbol of {4^12^.6^3^} (see **pcu** in Figure 4). Oddly **pcu** is rare, with only one instance ODIVUT in which cobalt atoms serve as all 6-connected nodes while bridging title ligands and water serve as edges.
ODIVUT[128]

### 5.6. ths

Topology **ths** has the point symbol of {10^3^} which is a uninodal net containing only 3-connected nodes (see **ths** in Figure 4). Both the ligands and metal atoms serve as the 3-connected nodes.
JODGEP[158]KAVGEU[159]

### 5.7. rtl

**Figure 5:**
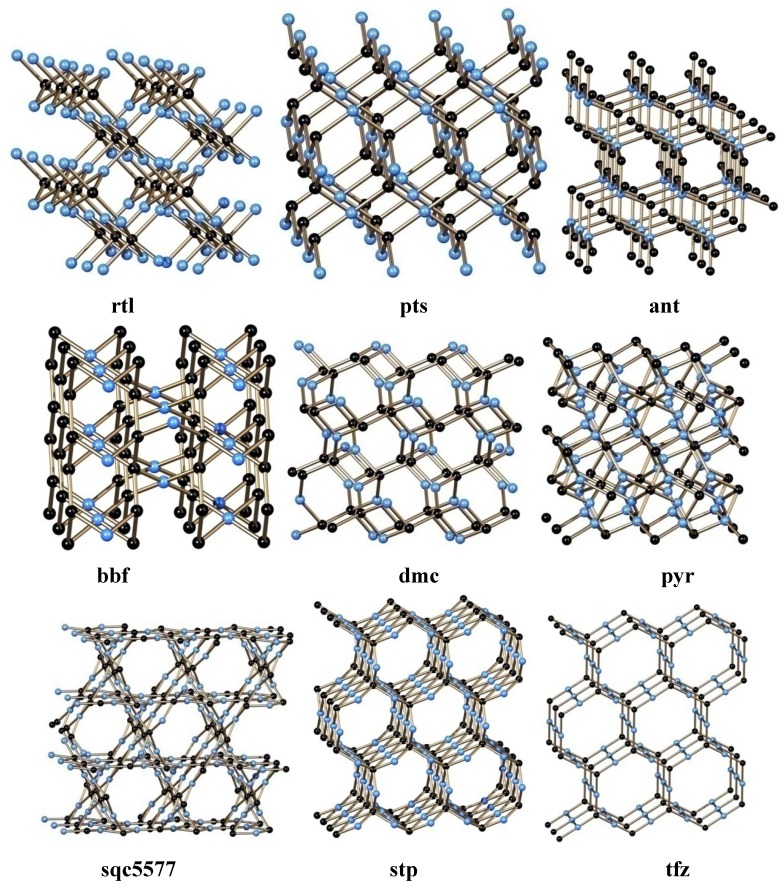
Topology of the binodal three-dimensional networks.

Topology **rtl** has the point symbol of {4.6^2^}_2_{4^2^.6^10^.8^3^}. It contains 3-connected nodes and 6-connected nodes (see **rtl** in Figure 5). All **rtl** structures also use tetrazole-containing ligands as 3-connected nodes exclusively, and metal atoms as 6-connected nodes.
JOJHUM[45]JOJJAU[45]JOJJIC[45]

### 5.8. pts

Topology **pts** has the point symbol of {4^2^.8^4^} and vertex symbol of [4.4.8(7).8(7).8(7).8(7)] [4.4.8(2).8(2).8(8).8(8)] (see **pts** in Figure 5). The vertex symbol shows that there are two different 4-connected nodes in the framework. All **pts** structures have tetrazole-containing ligands since they have enough coordination atoms to form the 4-connected nodes. Metal atoms form the other kind of 4-connected nodes.
LARBOW[32]REHRAY[33]LARBOW01[35]

### 5.9. ant

Topology **ant** contains 3-connected nodes and 6-connected nodes and has the point symbol of {4^2^.6}_2_{4^4^.6^2^.8^8^.10} (see **ant** in Figure 5). JOJJEY is the only entry that has the **ant** topology. Zinc atoms are its 6-connected nodes while title ligands are the 3-connected nodes.
JOJJEY[45]

### 5.10. bbf

There are two different kinds of 4-connected vertexes in topology **bbf** whose point symbol is {6^4^.8^2^}{6^6^}_2_ (see **bbf** in Figure 5). UHUNEQ, in which copper atoms and titled ligands represent different 4-connected nodes, is the only entry that possesses **bbf** topology.
UHUNEQ[160]

### 5.11. dmc

3-connected nodes and 4-connected nodes are presents in **dmc** topology whose point symbol is {4.8^2^}{4.8^5^} (see **dmc** in Figure 5). The only instance of **dmc** in this research is XOHPOA, whose 4-connected nodes are cadmium atoms and 3-connected nodes are title ligands. 2-connected pillars are also contained in the structure.
XOHPOA[161]

### 5.12. pyr

**pyr** is a 3,6-connected binodal net with point symbol of {6^12^.8^3^}{6^3^}_2_ (see **pyr** in Figure 5). QEYWUN is the only structure here that has a **pyr** topology. Cadmium atoms serve as 6-connected node and title ligands as 3-connected nodes.
QEYWUN[155]

### 5.13. sqc5577

**sqc5577** is a 4,4-connected binodal net in *epinet.ttd* database in TTD collection with the point symbol of {4^2^.6^2^.8^2^}{4^2^.6^3^.8} (see **sqc5577 in** Figure 5). Both cadmium atoms and title ligands serve as 4-connected nodes of a different type.
TONLOY[162]

### 5.14. stp

**stp** is a 4,6-connected binodal net with a point symbol of {4^4^.6^2^}_3_{4^9^.6^6^}_2_ (see **stp** in Figure 5). Both title ligands and carbonate ions in AGARUW are 4-connected nodes and the lanthanum atoms are 6-connected nodes. It is a porous structure.
AGARUW[163]

### 5.15. tfz

**tfz** is a 3,4-connected binodal net. Its point symbol is {6^3^}_2_{6^4^.8.10}_3_ (see **tfz** in Figure 5). Like KAVGAQ, **tfz** is formed by connection of edge center of neighboring **hcb** layers, but has a higher symmetry.

In REJLOI, title ligands are the 3-connected nodes and cobalt atoms are the 4-connected nodes.
REJLOI[164]

### 5.16. KAVGAQ

**Figure 6:**
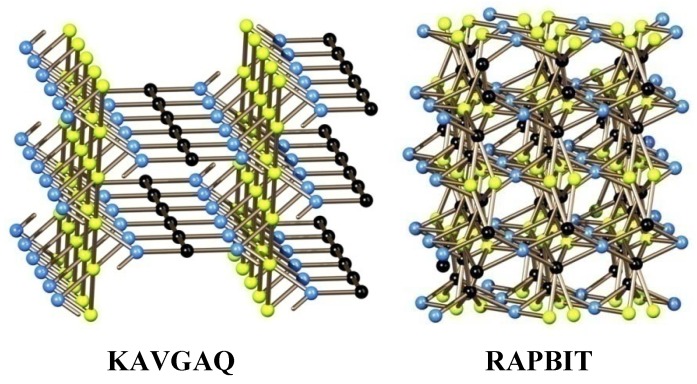
Topology of the 3-nodal three-dimensional networks.

**KAVGAQ** is a unique topology only present in coordination polymers with an imidazole-4,5-dicarboxylic acid ligand. It can be recognized by the central points of edges of **hcb** layers connected by pillars (see **KAVGAQ** in Figure 6). Its point symbol is {6^3^}_2_{6^4^.10^2^}{6^4^.8^2^}_2_. One kind of 3-connected node and three kinds of 4-connected node are present. In all instances, title ligands serve as 3-connected nodes and metal atoms as 4-connected nodes.
KAVGAQ[159]REJLEY[164]REJLIC[164]XECBUD[165]

### 5.17. RAPBIT

**RAPBIT** is a unique 5, 6, 6-connected 3-nodal net whose point symbol is {3.4^3^.5^6^.6^5^}_2_{3.4^6^.5^3^}_2_{3^2^.4^2^.5^2^.6^4^.7^4^.8} (see **RAPBIT** in Figure 6). Cadmium atoms are separated into two classes of 6-connected nodes while title ligands are the 5-connected nodes.
RAPBIT[153]

### 5.18. New three-dimensional topologies

Twenty four new topologies which are not present in the TTD collection are listed below (Shown in Table 2). 

## 6. Summary and Conclusions

This review shows that a number of coordination sites provided by the azole-containing carboxylic acid ligand are readily available to bind to metal ions as polydentate O and N donors and these multifunctional ligands can provide a variety of the topology patterns in the resulting infinite metal-organic coordination networks (MOCNs). The diverse coordination modes of diazole, triazole and tetrazole-containing carboxylic acids and the various topology patterns in the one, two, and three-dimensional metal-organic coordination polymers enrich the fields of research in the coordination and structural chemistry of these compounds, and contribute plentiful novel MOFs materials with better practical value as supporting carriers, in gas sorption and magnetic, optic or electronic applications.

## Figures and Tables

**Table 1:** New Two-Dimensional Topologies.

CSD Refcode	Topology Demonstration	Vertex Symbol*	Nodal Connectivity	Node Types
UFETEF[143]	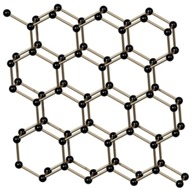	{4^3^.6^3^}	4-c	uninodal
BIZVEM[144] GIZSIS[145] RANBAK[146]	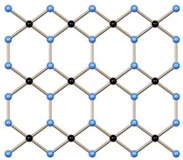	{4.6^2^}_2_{4.6.4.6}	3, 4-c	2-nodal
LILYIP[147] (Considering an octacobalt cluster as a single node)	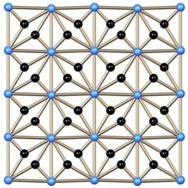	{3^2^.4}_2_{3^6^.4.3^2^.4}	3,10-c	2-nodal
JOCGIS[148]	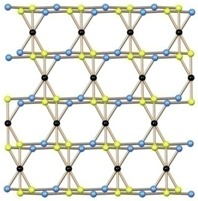	{3.4.5.6^2^.7}_2_{3.6.7}_2_{3^2^.4^2^.5^2^.6^2^.7^6^.8}*	3,4,6-c	3-nodal
KOBHEP[40]	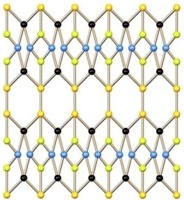	{4^3^.6}{4^4^}{4^3^}{4^2^,6^2^}	3,4,4,4-c	4-nodal
KOBHAL[40]	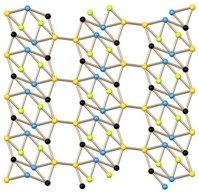	{3.4^2^.6}{3^2^.4^3^}{3.4^2^}{3^2^.4.6^2^}	3,4,5,5-c	4-nodal

* Symbols annotated with asterisk are point symbols (Schläfli symbol).

**Table 2:** New Three-Dimensional Topologies.

CSD Refcode	Topology Demonstration	Point Symbol (Schläfli Symbol)	Nodal Connectivity	Node Types
UFETAB[143]	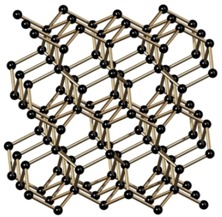	{6^6^}	4,4-c	uninodal
POLDOK[129] POLDUQ[129]	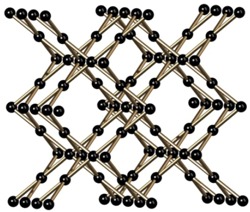	{4^2^.8^4^}	4-c	uninodal
MOFTIL[46] MOFTIL01[47]	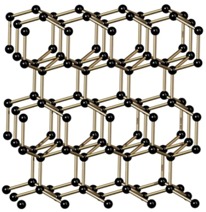	{6^6^}	4,4-c	uninodal
ACUXAY[166]	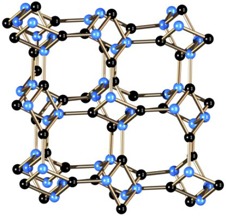	{4.8^2^}{4.8^5^}	3,4-c	binodal
BOHVEA[44] BOHVIE[44]	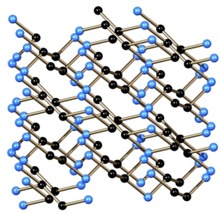	{4.8^2^.10^3^}{4.8^2^}	3,4-c	2-nodal
XECBOX[165]	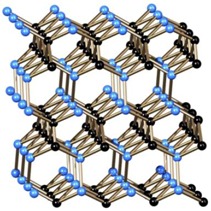	{10^3^}	3,3-c	uninodal
MEVBIZ[151]	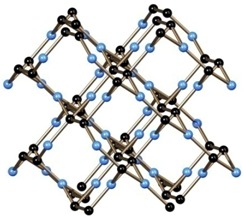	{4.6.8}{4.6^2^.8.10^2^}	3,4-c	2-nodal
PEXSOB[93] PEXSOB01[167]	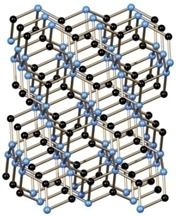	{4.6^2^}{4.6^7^.8^2^}	3,5-c	2-nodal
QEYWOH[155]	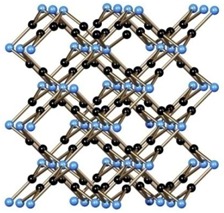	{4^2^.6}_2_{4^4^.6^2^.8^7^.10^2^}	3,6-c	2-nodal
YELYUK[92]	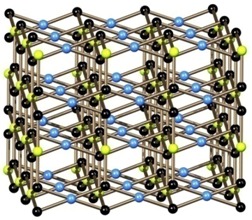	{4^2^.8^4^}{4^6^.6^6^.8^3^}{4^8^.6^2^}_2_	4,5,6-c	3-nodal
GIDKOU[168]	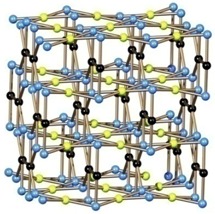	{4^2^.5^2^.7^2^.8^4^} {4^2^.5^2^.7^2^}_2_{4^2^.8^4^}	4,4,4,5-c	3-nodal
UFARUP[169]	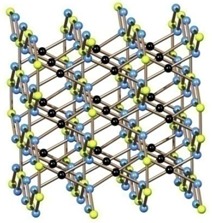	{4^2^.5^2^.7^2^.8^4^} {4^2^.5^2^.7^2^}_2_{4^2^.8^4^}	4,4,4,5,5-c	3-nodal
UFASAW[169]	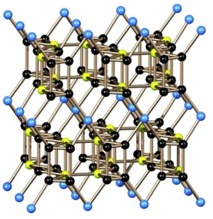	{4.5.6^3^.7}_4_ {4^2^.5^2^.6^4^.7^2^}_2_{5^2^.8^4^}	4,4,4,5,5-c	3-nodal
DIXVUC[39]	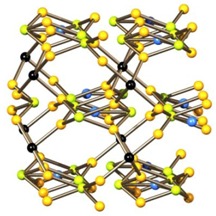	{4.8^2^}_6_{4^3^}{4^6^.6^4^}_3_{8^12^.12^3^}	3,3,5,6-c	4-nodal
CETGEO[170]	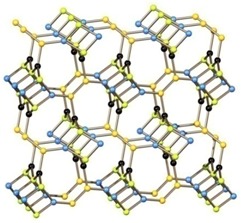	{4.8^4^.10}{4.9^2^}{8.9^2^}{9^3^}	3,3,3,4-c	4-nodal
KOCWEF[41]	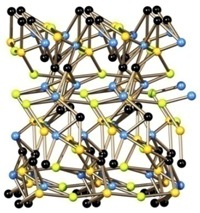	{3.4.6^2^.7^2^}{3.4^3^.5^2^.6.7^2^.8}{3^2^.4^4^.5^2^.6^2^}{3^2^.4^5^.5.6^4^.7^3^}	4,4,5,5,5,5,6,6-c	4-nodal
WIJDAV[171]	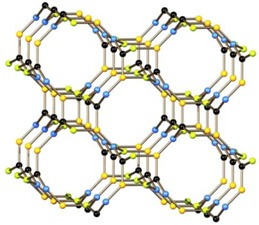	{6.10^2^}{6.8.10}_2_{8.10^2^}	3,3,3,3-c	4-nodal
LIZWEX[49]	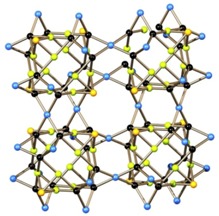	{4.6.8^2^.10^2^}_6_{4^2^.6}_6_ {4^3^}_2_{6^2^.8^4^}_3_	3,3,4,4-c	4-nodal
XECBIR[165]	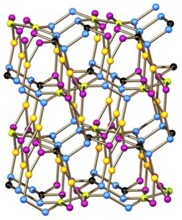	{4.6^2^.8^2^.9}_2_{4.8^2^}_2_{4^2^.5^2^.6.7^2^.8^3^}{4^2^.5^2^.7^2^}_2_ {4^2^.6.8^3^}	3,4,4,4,5-c	5-nodal
KOLWUE[172]	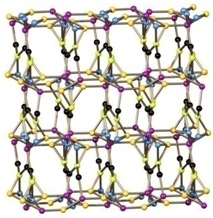	{4.6^2^}{4.6^4^.8^4^.10}{4.6^4^.8}{4.8^2^}{6^3^}	3,3,3,4,5-c	5-nodal
KEPMIB[173]	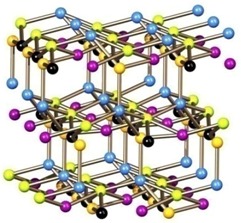	{4.6.8^3^.10}{4.6.8}_2_{4^3^.6^6^.8^6^}{4^3^.8^3^}	3,3,4,4,6-c	5-nodal
GIKBOS[174]	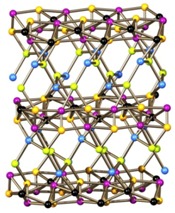	{4.6.8}{4^2^.6^3^.8}{4^3^.6^2^.8}{4^4^.6^5^.8^6^}{4^5^.6^4^.8}	3,4,4,5,6-c	5-nodal
YELYOE[92]	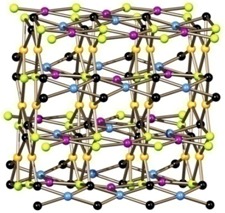	{4^2^.5^2^.6^2^}_2_{4^2^.5^2^.7^2^.8^4^} {4^2^.5^4^.8^4^}{4^3^.5.6^2^.7^2^.8^2^}_2_ {4^7^.5.6^2^}_2_	4,5,5,5,5-c	5-nodal
NEVHEC[175] NEVHIG[175] NEVHOM[175] NEVHUS[175] NEVJAA[175] NEVJEE[175] NEVJII[175] NEVJOO[175] NEVJUU[175]	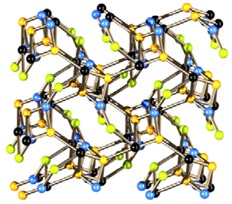	{4.8^2^}{4.8^5^}{4^2^.8^8^}{4^6^}	3,4,4,5-c	4-nodal

## References

[1] Caulder D.L., Raymond K.N. (1999). Supermolecules by design. Acc. Chem. Res..

[2] Seidel S.R., Stang P.J. (2002). High-symmetry coordination cages via self-assembly. Acc. Chem. Res..

[3] Yaghi O.M., O’Keeffe M., Ockwig N.W., Chae H.K., Eddaoudi M., Kim J. (2003). Reticular synthesis and the design of new materials. Nature.

[4] Kesanli B., Lin W.B. (2003). Chiral porous coordination networks: Rational design and applications in enantioselective processes. Coord. Chem. Rev..

[5] Frey G., Mellot-Draznieks C., Serre C., Millange F. (2005). Crystallized frameworks with giant pores: Are there limits to the possible?. Acc. Chem. Res..

[6] Bradshaw D., Claridge J.B., Cussen E.J., Prior T.J., Rosseinsky M.J. (2005). Design, chirality, and flexibility in nanoporous molecule-based materials. Acc. Chem. Res..

[7] Fujita M., Tominaga M., Hori A., Therrien B. (2005). Coordination assemblies from a Pd(II)-cornered square complex. Acc. Chem. Res..

[8] Robin A.Y., Fromm K.M. (2006). Coordination polymer networks with O- and N-donors: what they are, why and how they are made. Coord. Chem. Rev..

[9] Wei Y.Q., Yu Y.F., Wu K.C. (2007). Highly stable diamondoid network coordination polymer [Mn(NCP)_2_]*_n_* with notable NLO, magnetic, and luminescence properties. Cryst. Growth Des..

[10] Su C.Y., Smith M.D., Goforth A.M., Zur Loye H. (2004). A Three-dimensional, noninterpenetrating metal-organic framework with the moganite topology: A simple (4^2^.6^2^.8^2^)(4.6^4^.8)^2^ net containing two kinds of topologically nonequivalent points. Inorg. Chem..

[11] Kitagawa S., Masaoka S. (2003). Metal complexes of hexaazatriphenylene (hat) and its derivatives - from oligonuclear complexes to coordination polymers. Coord. Chem. Rev..

[12] Bu X.H., Tong M.L., Chang H.C., Kitagawa S., Batten S.R. (2004). A neutral 3D copper coordination polymer showing 1D open channels and the first interpenetrating NbO-type network. Angew. Chem. Int. Ed..

[13] Yong G.P., Wang Z.Y., Cui Y. (2004). Synthesis, structural characterization and properties of copper(Ⅱ) and zinc(Ⅱ) coordination polymers with a new bridging chelating ligand. Eur. J. Inorg. Chem..

[14] Chang F., Wang Z.M., Sun H.L., Wen G.H., Zhang X.X. (2005). [Cu_2_(bpdado)_2_(H_2_O)_2_]·H_2_O}_n_: A 1D nanotubular coordination polymer with wall made of edge-sharing hexagons, where bpdado=2,2′-bipyridine-3,3′-dicarboxylate-1,1′-dioxide. Dalton Trans..

[15] Zaworotko M.J. (1998). From disymmetric molecules to chiral polymers: a new twist for supramolecular synthesis?. Angew. Chem., Int. Ed..

[16] Cao R., Sun D.F., Liang Y.C., Hong M.C., Tatsumi K., Shi Q. (2002). Syntheses and characterizations of three-dimensional channel-like polymeric lanthanide complexes constructed by 1,2,4,5-benzenetetracarboxylic acid. Inorg. Chem..

[17] Liang Y.C., Cao R., Su W.P., Hong M.C., Zhang W.J. (2000). Syntheses, structures, and magnetic properties of two gadolinium(III)-copper(II) coordination polymers by a hydrothermal reaction. Angew. Chem., Int. Ed..

[18] Chapman M.E., Ayyappan P., Foxman B.M., Yee G.T., Lin W.B. (2001). Synthesis, x-ray structures, and magnetic properties of copper(II) pyridinecarboxylate coordination networks. Cryst. Growth Des..

[19] Lu J.Y., Schauss V. (2001). Crystal engineering of a three-dimensional coordination polymer based on both covalent and O–H···O hydrogen bonding interactions of bifunctional ligands. CrystEngComm..

[20] Noro S., Kitagawa S., Yamashita M., Wada T. (2002). New microporous coordination polymer affording guest-coordination sites at channel walls. Chem. Commun..

[21] Lu J.Y., Babb A.M. (2001). An unprecedented interpenetrating structure with two covalent-bonded open-framework of different dimensionality. Chem. Commun..

[22] Tong M.L., Li L.J., Mochizuki K., Chang H.C., Chen X.M., Li Y., Kitagawa S. (2003). A novel three-dimensional coordination polymer constructed with mixed-valence dimeric copper(I,II) units. Chem. Commun..

[23] Kang Y., Yao Y.G., Qin Y.Y., Zhang J., Chen Y.B., Li Z.J., Wen Y.H., Cheng J.K., Hu R.F. (2004). A novel ligand-unsupported 3D framework polymer of trimeric copper(I) and its NLO property. Chem. Commun..

[24] Eubank J.F., Walsh R.D., Eddaoudi M. (2005). Terminal co-ligand directed synthesis of a neutral, non-interpenetrated (10,3)-*a* metal–organic framework. Chem. Commun..

[25] Lou B.Y., Jiang F.L., Wu B.L., Yuan D.Q., Hong M.C. (2006). From helical array to porous architecture: exploring the use of side chains of amino acids to engineer 1D infinite coordination polymeric chain into porous frameworks. Cryst. Growth Des..

[26] Zeng Y.F., Liu F.C., Zhao J.P., Cai S., Bu X.H., Ribas J. (2006). An azido–metal–isonicotinate complex showing long-range ordered ferromagnetic interaction: synthesis, structure and magnetic properties. Chem. Commun..

[27] Liu F.C., Zeng Y.F., Zhao J.P., Hu B.W., Hu X., Ribas J., Bu X.H. (2009). Novel lanthanide–azido complexes: Hydrothermal syntheses, structures and magnetic properties. Dalton Trans..

[28] Hu X., Zeng Y.F., Chen Z., Saudo E.C., Liu F.C., Ribas J., Bu X.H. (2009). 3d−4f coordination polymers containing alternating EE/EO azido chain synthesized by synergistic coordination of lanthanide and transition metal ions. Cryst. Growth Des..

[29] Batten S.R., Robson R. (1998). Interpenetrating nets: ordered, periodic entanglement. Angew. Chem. Int. Ed..

[30] Qu Z.R., Zhao H., Wang X.S., Li Y.H., Song Y.M., Liu Y.J, Ye Q., Xiong R.G., Abrahams B.F., Xue Z.L., You X.Z. (2003). Homochiral Zn and Cd coordination polymers containing amino acid−tetrazole ligands. Inorg. Chem..

[31] He F., Tong M.L., Yu X.L., Chen X.M. (2005). Controlled aggregation of heterometallic nanoscale Cu_12_Ln_6_ clusters (Ln = Gd^III^ or Nd^III^) into 2D coordination polymers. Inorg. Chem..

[32] Wang X.S., Huang X.F., Xiong R.G. (2005). An unexpected intermediate or precipitate-novel 3D Cd-coordination polymer formed in the preparation of 5-substituted 1H-tetrazoles from nitrile in water. Chin. J. Inorg. Chem..

[33] Ye Q., Song Y.M., Wang G.X., Chen K., Fu D.W., Chan W.H., Zhu J.S., Huang S.P., Xiong R.G. (2006). Ferroelectric metal-organic framework with a high dielectric constant. J. Am. Chem. Soc..

[34] Rodriguez-Dieguez A., Colacio E. (2006). [Zn_n_(polyox)(pmtz)_n_]: The first polyoxalate-containing coordination polymer from an unforeseen chemical rearrangement of 5-pyrimidyltetrazole under hydrothermal conditions. Chem. Commun..

[35] Jiang T., Zhao Y.F., Zhang X.M. (2007). Blue-green photoluminescent 5-and 10-connected metal 5-(4′-carboxy-phenyl)tetrazolate coordination polymers. Inorg. Chem. Commun..

[36] Yang G.W., Li Q.Y., Wang J., Yuan R.X., Xie J.M. (2007). New Cu^II^ and Cd^II^ coordination polymers employing 5-[N-acetato(4-pyridyl)] tetrazolate as a bridging ligand. Chin. J. Inorg. Chem..

[37] Fu D.W., Zhao H. (2007). Intermediate captured in the reaction of synthesizing valartan analogue (I). Chin. J. Inorg. Chem..

[38] Huang X.H., Sheng T.L., Xiang S.C., Fu R.B., Hu S.M., Li Y.M., Wu X.T. (2007). Synthesis, structure and luminescence of a novel 2D cadmium coordination polymer with a ligand generated *in situ*. Chin. J. Struct. Chem..

[39] Bai Y.L., Tao J., Huang R.B., Zheng L.S., Zheng S.L., Oshida K., Einaga Y. (2008). Pressure effects and mössbauer spectroscopic studies on a 3D mixed-valence iron spin-crossover complex with NiAs topology. Chem. Commun..

[40] Yu Q., Zhang X.Q., Bian H.D., Liang H., Zhao B., Yan S.P., Liao D.Z. (2008). pH-Dependent Cu(II) coordination polymers with tetrazole-1-acetic acid: synthesis, crystal structures, EPR and magnetic properties. Cryst. Growth Des..

[41] Yu Z.P., Xie Y., Wang S.J., Yong G.P., Wang Z.Y. (2008). Synthesis, crystal structures and optical properties of two coordination polymers from 4-(1H-tetrazol-5-yl) benzoic acid. Inorg. Chem. Commun..

[42] Dong W.W., Zhao J., Xu L. (2008). Syntheses, crystal structure and properties of two novel coordination polymers with the flexible tetrazole-1-acetic acid (Htza). J. Solid State Chem..

[43] Yang G.W., Li Q.Y., Zhou Y., Sha P., Ma Y.S., Yuan R.X. (2008). Mn and Cu-Na coordination compounds containing the tetrazole-5-acetato anion (tza) ligands. Inorg. Chem. Commun..

[44] Jia Q.X., Wang Y.Q., Yue Q., Wang Q.L., Gao E.Q. (2008). Isomorphous Co-II and Mn-II materials of tetrazolate-5-carboxylate with an unprecedented self-penetrating net and distinct magnetic behaviours. Chem. Commun..

[45] Dong W.W., Zhao J., Xu L. (2008). Remarkable structural transformation of [Zn(tza)(2)] during recrystallization, syntheses and crystal structures of [M(tza)(2)] (M = Zn, Cd, Mn, Co; Htza = tetrazole-1-acetic acid. Cryst. Growth Des..

[46] Zhang J.Y., Wang Y.Q., Peng H.Q., Cheng A.L., Gao E.Q. (2008). Synthesis, structure, and photoluminescence of a zinc(II) coordination polymer with 4-(tetrazol-5-yl)benzoate. Struct. Chem..

[47] Li Y., Xu G., Zou W.Q., Wang M.S., Zheng F.K., Wu M.F., Zeng H.Y., Guo G.C., Huang J.S. (2008). A novel metal-organic network with high thermal stability: Nonlinear optical and photoluminescent properties. Inorg. Chem..

[48] Yang G.W., Li Q.Y., Zhou Y., Gu G.Q., Ma Y.S., Yuan R.X. (2008). Two copper(II) coordination polymers containing atza ligand [atza = 5-aminotetrazole-1-acetato]. Inorg. Chem. Commun..

[49] Nouar F., Eubank J.F., Bousquet T., Wojtas L., Zaworotko M.J., Eddaoudi M. (2008). Supermolecular building blocks (SBBs) for the design and synthesis of highly porous metal-organic frameworks. J. Am. Chem. Soc..

[50] Li Q.Y., Yang G.W., Yuan R.X, Wang J.P., Cui P.F. (2008). Bis(5-aminotetrazole-1-acetato-*κ*O)tetraaquacobalt(II) and *catena*-Poly[[cadmium(II)]-bis(*μ*-5-aminotetrazole-1-acetato-*κ*^3^N^4^:O,O′)]. Acta Crystallogr..

[51] Keene T.D., Deng Y.H., Li F.G., Ding Y.F., Wu B., Liu S.X., Ambrus C., Waldmann O., Decurtins S., Yang X.J. (2009). Magnetostructural investigations into an S = 1/2 sheet and a tetranuclear butterfly cluster. Inorg. Chim. Acta.

[52] Aromi G., Roubeau O., Helliwell M., Teat S.J., Winpenny R.E.P. (2003). Novel topologies in Ni^II^ cluster chemistry: Incorporation of alkaline-earth metals in the new [Ni^II^_6_Mg^II^_2_] and [Ni^II^_8_M^II^](M = Sr, Ba) cages. Dalton Trans..

[53] Liu Y.L., Kravtsov V., Walsh R.D., Poddar P., Srikanth H., Eddaoudi M. (2004). Directed assembly of metal–organic cubes from deliberately predesigned molecular building blocks. Chem. Commun..

[54] Zou R.Q., Jiang L., Senoh H., Takeichi N., Xu Q. (2005). Rational assembly of a 3D metal–organic framework for gas adsorption with predesigned cubic building blocks and 1D open channels. Chem. Commun..

[55] Xu Q., Zou R.Q., Zhong R.Q., Kachi-Terajima C., Takamizawa S. (2008). Cubic metal−organic polyhedrons of Nickel(II) imidazole dicarboxylate depositing protons or alkali metal ions. Cryst. Growth Des..

[56] Allen F. (2002). The Cambridge Structure Database: A quarter of a million crystal structures and rising. Acta Crystallogr..

[57] Allen F., Motherwell W.D.S. (2002). Applications of the Cambridge Structural Database in organic and crystal chemistry. Acta Crystallogr..

[58] Zorkii P.M., Oleinikov P.N. (2001). Crystal-chemical classes of “Cambridge” crystal structures: Statistical analysis of topology. J. Struct. Chem..

[59] Blatov V.A. (2006). Multipurpose crystallochemical analysis with the program package TOPOS. IUCr CompComm Newsletter.

[60] Peresypkina E.V., Blatov V.A. (2000). Topology of molecular packings in organic crystals. Acta Crystallogr.

[61] Wells A.F. (1979). Further studies of three-dimensional nets.

[62] Smith J.V. (1978). Enumeration of 4-connected 3-dimensional nets and classification of framework silicates, II. Perpendicular and near-perpendicular linkages from 4.^82^, 3.1^22^ and 4.6.12 nets. Amer. Mineral..

[63] O’Keeffe M., Peskov M.A., Ramsden S.J., Yaghi O.M. (2008). The reticular chemistry structure resource (RCSR) database of, and symbols for, crystal nets. Acc. Chem. Res..

[64] Colacio E., Dominguez-Vera J.M., Ghazi M., Kivekas R., Klinga M., Moreno J.M. (1999). Singly anti-anti carboxylate-bridged zig-zag chain complexes from a carboxylate-containing tridentate schiff base ligand and M(hfac)_2_ [M = Mn^II^, Ni^II^, and Cu^II^]: Synthesis, crystal structure, and magnetic properties. Eur. J. Inorg. Chem..

[65] Liu Y.Y. (2007). *catena*-Poly[[triaquamanganese(II)]-*μ*-1,2,4-triazole-3,5-dicarboxylato-*κ*^3^O^3^:N^4^,O^5^]. Acta Crystallogr..

[66] Sun Y.Y., Zhang Y.W., Zhang G., Cheng L. (2008). *catena*-Poly[[triaquazinc(II)]-*μ*-1H-1,2,4-triazole-3,5-dicarboxylato]. Acta Crystallogr..

[67] King P., Clerac R., Anson C.E., Powell A.K. (2004). The building block approach to extended solids: 3,5-pyrazoledicarboxylate coordination compounds of increasing dimensionality. Dalton Trans..

[68] Santillan G.A., Carrano C.J. (2008). Cobalt, Zinc, and Nickel complexes of a diatopic heteroscorpionate ligand: building blocks for coordination polymers. Inorg. Chem..

[69] Hammes B.S., Kieber-Emmons M.T., Letizia J.A., Shirin Z., Carrano C.J., Zakharov L.N., Rheingold A.L. (2003). Synthesis and characterization of several zinc(II) complexes containing the bulky heteroscorpionate ligand bis(5-*tert*-butyl-3-methylpyrazol-2-yl)acetate: Relevance to the resting states of the zinc(II) enzymes thermolysin and carboxypeptidase A. Inorg. Chim. Acta.

[70] Dou Q.Q., He Y.K., Zhang L.T., Han Z.B. (2007). *catena*-Poly[4,4′-bipyridinium [bis(*μ*_3_-pyrazole-3,5-dicarboxylato-*κ*^5^O^5^,N^1^:N^2^,O^3^:O^3^)dicopper(II)]]. Acta Crystallogr..

[71] An C.X., Lu Y.C., Shang Z.F., Zhang Z.H. (2008). Syntheses and crystal structures of the metal complexes based on pyrazolecarboxylic acid ligands. Inorg. Chim. Acta.

[72] Tian J.L., Yan S.P., Liao D.Z., Jiang Z.H., Cheng P. (2003). Syntheses, structures and properties of two one-dimensional chain complexes: [Mn(Hpdc)(H_2_O)_2_]*_n_* and [Cu_2_(Hpdc)_2_][4,4^′^-dpdo] (Hpdc=3,5-pyrazoledicarboxylic acid group, dpdo=4,4^′^-dipyridyl-*N*,*N*^′^-dioxide hydrate). Inorg. Chem. Commun..

[73] Branzea D.G., Guerri A., Fabelo O., Ruiz-Perez C., Chamoreau L.-M., Sangregorio C., Caneschi A., Andruh M. (2008). Heterobinuclear complexes as tectons in designing coordination polymers. Cryst. Growth Des..

[74] Gu C.S., Gao S., Huo L.H., Zhao H., Zhao J.G. (2004). *catena*-Poly[[(1,10-phenanthroline-*κ*^2^N,N′)copper(II)]-*μ*-4-carboxyimidazole-5-carboxylato(2-)-*κ*^4^N,O:N′,O′]. Acta Crystallogr..

[75] Wang X., Qin C., Wang E., Xu L. (2005). New one-dimensional imidazole-bridged cadmium(II) coordination polymers-syntheses, crystal structures and photoluminescence. J. Mol. Struct..

[76] Mijangos E., Costa J.S., Roubeau O., Teat S.J., Gamez P., Reedijk J., Gasque L. (2008). Self-assembly of an infinite Copper(II) chiral metallohelicate. Cryst. Growth Des..

[77] Hao L.J., Bao Z.M., Yu T.L. (2007). *catena*-Poly[[(2,2-bipyridine)cobalt(II)]-*μ*-imidazole-4,5-dicarboxylato]. Acta Crystallogr..

[78] Chen H.M., Yang S.P., Zhang F., Yu X.B. (2003). Synthesis, crystal structure and properties of aquacopper(II) N-[(1-Methylimidazole-2-yl) methylene]-β-alaninate hexafluoraphosphate and copper(II)[N-(1-methylimidaz ole-2-yl) methyl-β-alanine superchlorate. Synth. React. Inorg. Met. Org. Chem..

[79] Gao S., Liu J.W., Huo L.H. (2005). *catena*-Poly[[aquacadmium(II)]bis(*μ*-4,5-diphenyl-1H-imidazole-1-acetate)- *κ*^3^N:O,O′; *κ*^3^O,O′:N]. Acta Crystallogr..

[80] Long L.S., Yang S.P., Tong Y.X., Mao Z.W., Chen X.M., Ji L.N. (1999). Synthesis, crystal structures and properties of copper(II) complexes of Schiff base derivatives containing imidazole and β-alanine groups. J. Chem. Soc. Dalton Trans..

[81] Landaverry Y.R., White K.N., Olmstead M.M., Einarsdottir O., Konopelski J.P. (2006). Cytochrome c oxidase active site mimics: New ligands for copper and an unexpected oxidative c-c bond formation. Heterocycles.

[82] Deng Q.J., Zeng M.H., Liang H., Ng S.W., Huang K.L. (2006). *catena-*Poly[[[diaquamanganese(II)]bis(*μ*-1H-benzimidazole-5-carboxylato)-*κ*^2^N^3^:O; *κ*^2^O:N^3^] dihydrate]. Acta Crystallogr..

[83] Wang L., Cai J.W., Mao Z.W., Feng X.L., Huang J.W. (2004). Dinickel complexes bridged by unusual (N,O,O′)-coordinated α-amino acids: syntheses, structural characterization and magnetic properties. Transit. Metal Chem..

[84] Gao S., Gu C.S., Huo L.H., Zhao H., Zhao J.G. (2004). *catena*-Poly[[(1,10-phenanthroline-*κ*^2^N,N′)cadmium(II)]-*μ*-imidazole-4,5-dicarboxylato-*κ*^4^N,O:N,O′]. Acta Crystallogr..

[85] Liu Z., Chen Y., Liu P., Wang J., Huang M.H. (2005). Cadmium(II) and cobalt(II) complexes generated from benzimidazole-5-carboxylate: Self-assembly by hydrogen bonding and *π*–*π* interactions. J. Solid State Chem..

[86] Mahata P., Natarajan S. (2005). Pyridine- and imidazoledicarboxylates of zinc: Hydrothermal synthesis, structure, and properties. Eur. J. Inorg. Chem..

[87] Colacio E., Ghazi M., Kivekas R., Moreno J.M. (2000). Helical-chain copper(II) complexes and a cyclic tetranuclear copper(II) complex with single syn−anti carboxylate bridges and ferromagnetic exchange interactions. Inorg.Chem..

[88] Zeng M.H., Zhou Y.L., Ng S.W. (2006). *catena*-Poly[[diaqua[(Z)-3-(1H-benzimidazol-2-yl)prop-2-enoato-*κ*^2^N,O]cobalt(II)]-*μ*-(Z)-3-(1H-benzimidazol-2-yl)prop-2-enoato-*κ*^2^O:O′]. Acta Crystallogr..

[89] Fan J., Zhang Y.A., Okamura T., Zou Z.H., Ueyama N., Sun W.Y. (2001). Synthesis and crystal structure of a one-dimensional coordination polymer of nickel(II) with 4^′^-(imidazol-1-ylmethyl)benzoate anion. Inorg. Chem. Commun..

[90] Qin C., Wang E.B. (2007). *catena*-Poly[[aqua(4,4′-bipyridine-*κ*N)manganese(II)]-*μ*-imidazole-4,5-dicarboxylato-*κ*^4^N^3^,O^4^:O^4′^,O^5^]. Acta Crystallogr..

[91] Fang R.Q., Zhang X.M. (2006). Diversity of coordination architecture of metal 4,5-dicarboxyimidazole. Inorg. Chem..

[92] Sun Y.Q., Zhang J., Yang G.Y. (2004). *catena*-Poly[[diaquacadmium(II)]-μ-5-carboxyimidazole-4-carboxyl-ato-κ^4^N^1^,O^5^:O^4^,N^3^]. Acta Crystallogr..

[93] Yao Y.L., Che Y.X., Zheng J.M. (2008). Structural and fluorescent characterizations of one-and two-dimensional Cd(II)metal-organic frameworks. Inorg. Chem. Commun..

[94] Guo Z.G., Cao R., Li X.J., Yuan D.Q., Bi W.H., Zhu X.D., Li Y.F. (2007). A Series of cadmium(II) coordination polymers synthesized at different pH. Eur. J. Inorg. Chem..

[95] Bai Y.L., Tao J., Huang R.B., Zheng L.S. (2005). A three-dimensional supramolecular network built with the zigzag chain complex bis(5-carboxy-1*H*-imidazole-4-carboxylato)copper(II). Acta Crystallogr..

[96] Lin X.F. (2006). *catena*-Poly[[aqua(*μ*-5-carboxyimidazole-4-carboxylato-*κ*^4^N^3^,O^4^:N^1^,O^5^)zinc(II)] hemi-hydrate]. Acta Crystallogr..

[97] Hao L.J., Yu T.L. (2007). *catena*-Poly[[(2,2′-bipyridine)nickel(II)]-*μ*-imidazole-4,5-dicarboxylato]. Acta Crystallogr..

[98] Bruijnincx P.C.A., Lutz M., den Breejen J.P., Spek A.L., van Koten G., Gebbink R.J.M.K. (2007). Zinc complexes of the biomimetic N,N,O ligand family of substituted 3,3-bis(1-alkylimidazol-2-yl)propionates: The formation of oxalate from pyruvate. J. Biol. Inorg. Chem..

[99] Drozdzewski P., Pawlak B., Glowiak T. (2002). Unusual coordination behavior of imidazole-4-acetic acid. Synthesis, crystal structure and vibrational studies of one-dimensional co-ordination polymer of zinc(II) with two different ligand forms. Polyhedron.

[100] Li X.M., Dong Y.H., Wang Q.W., Liu B. (2007). *catena*-Poly[[(2,2′-bipyridine-*κ*^2^N,N′)zinc(II)]-*μ*-imidazole-4,5-dicarboxylato-*κ*^4^N^1^,O^5^:N^3^,O^4^]. Acta Crystallogr..

[101] Li Z.F., Wang S.W., Zhang Q., Yu X.J. (2007). *catena*-Poly[[(2,2′-bipyridine-*κ*^2^N,N′)iron(II)]-*μ*-5-carboxy-4-carboxylatoimidazol-1-ido-*κ*^4^N^3^,O^4^:N^1^,O^5^]. Acta Crystallogr..

[102] Akhriff Y., Server-Carrio J., Sancho A., Garcia-Lozano J., Escriva E., Soto L. (2001). Two polymeric compounds built from mononuclear and tetrameric squarate−copper(II) complexes by deprotonation of 3,3-Bis(2-imidazolyl)propionic acid (HBIP). Synthesis, crystal structure, and magnetic characterization of [Cu(HBIP)(BIP)](C_4_O_4_)_1/2_·2H_2_O and [{Cu(BIP)(OH_2_)}_4_(μ-C_4_O_4_)](ClO_4_)_2_·4H_2_O. Inorg. Chem..

[103] Liu G.F., Ren Z.G., Chen Y., Liu D., Li H.X., Zhang Y., Lang J.P. (2008). Solvothermal synthesis, structure and luminescent properties of a new 3D coordination polymer [K_2_Cd(Htda)_2_]*_n_* (Htda = 1,2,3-triazole-4,5-dicarboxylate). Inorg. Chem. Commun..

[104] Chandrasekhar V., Thilagar P., Senapati T. (2007). Transition metal-assisted hydrolysis of pyrazole-appended organooxotin carboxylates accompanied by ligand transfer. Eur. J. Inorg. Chem..

[105] Abdeljalil E.F., Najib B.L., Abdelali K., Bali B.E., Bolte M. (2006). *catena*-Poly[[[(3,5-dimethyl-1H-pyrazole-*κ*N^2^) copper(II)]-*μ*-[(3,5-dimethyl-1H-pyrazol-1-yl)methylamino]acetato] nitrate monohydrate]. Acta Crystallogr..

[106] Gao S., Liu J.W., Huo L.H., Zhao H. (2004). *catena*-Poly[[(2,2′-bipyridine-^2^*N*,*N*′)cadmium(II)]--5-carboxyimidazole-4-carboxylato-^4^*N*^3^,*O*^4^:*N*^1^,*O*^5^]. Acta Crystallogr..

[107] Cheng A.L., Liu N., Zhang J.Y., Gao E.Q. (2007). Assembling the cage-based metal−organic network from a cubic metalloligand. Inorg. Chem..

[108] Yang J.H., Zheng S.L., Yu X.L., Chen X.M. (2004). Syntheses, structures, and photoluminescent properties of three silver(I) cluster-based coordination polymers with heteroaryldicarboxylate. Cryst. Growth Des..

[109] Han Z.B., Ma Y. (2006). Poly[di-*μ*_2_-aqua-*μ*-pyrazole-3,5-dicarboxylato-copper(II)]. Acta Crystallogr..

[110] Chen H., Ma C.B., Xiang S.C., Hu M.Q., Si Y.T., Chen C.N., Liu Q.T. (2008). Synthesis and characterization of vanadium(III) and vanadium(IV) polymers containing 3,5-pyrazoledicarboxylato. J. Coord. Chem..

[111] Kasuga N.C., Tsuruta S., Amano A., Nomiya K. (2007). Poly[(*μ*_3_-N-acetyl-L-histidinato-*κ*^4^N,O:O:O′)silver(I)]. Acta Crystallogr..

[112] Akhriff Y., Server-Carrio J., Sancho A., Garcia-Lozano J., Escriva E., Folgado J.V., Soto L. (1999). Synthesis, crystal structure, and magnetic properties of oxalato−copper(II) complexes with 3,3-bis(2-imidazolyl)propionic acid, an imidazole−carboxylate polyfunctional ligand: From mononuclear entities to ladder-like chains. Inorg. Chem..

[113] Han L., Gong Y.Q., Yuan D.Q., Hong M.C. (2006). Luminescent 2D supramolecular network constructed from tubular coordination polymer based on H-bonding and pi-pi interactions. J. Mol. Struct..

[114] Meng W.W., Chen J.X. (2008). Synthesis and crystal structures of new nickel(Ⅱ) and manganese(Ⅱ) coordination polymers containing 5-benzimidazolecarboxylate ligand. Chin. J. Inorg. Chem..

[115] Xu K., Yu L.P. (2009). *catena*-Poly[[di-*μ*-aqua-bis[aquacobalt(II)]]-bis(*μ*_3_-1H-benzimidazole-5,6-dicarboxylato). Acta Crystallogr..

[116] van Koningsbruggen P.J., van Hal J.W., Muller E., de Graaff V., G.Haasnoot J., Reedijk J. (1993). A novel type of twisted antiparallel double-chain structure with stacking between the two strands. Structure, synthesis and magnetic properties of [{[Cu_3_L_2_(dien)(H_2_O)_2_]·3H_2_O}_∞_][L = 1H-1,2,4-triazole-3,5-dicarboxylate(3–), dien = 3-Azapentane-1,5-diamine]. J. Chem. Soc. Dalton Trans..

[117] Wang J.J., Zhang B., Shu H.M., Du C.Q., Hu H.M. (2007). A two-dimensional coordination polymer containing linear trinuclear copper (II) clusters. Acta Crystallogr..

[118] Xu Y., Wang R.H., Lou B.Y., Han L., Hong M.C. (2004). Poly[iron(II)-di-*μ*-imidazole-4,5-dicarboxylato-*κ*^3^*N*^3^,*O*^4^:*O*^5^]. Acta Crystallogr..

[119] Guo Z.G., Yuan D.Q., Bi W.H., Li X.J., Cao R. (2006). A novel antiferromagnetic nickel coordination framework with 1-H-benzimidazole-5-carboxylic acid. J. Mol. Struct..

[120] Wang Y.T., Tang G.M., Qin D.W. (2006). Metal-controlled assembly tuning coordination polymers with flexible 2-(1H-imidazole-1-yl)acetic acid (Hima). Aust. J. Chem..

[121] Deng Q.J., Zeng M.H., Liang H., Huang K.L. (2006). Hydrothermal synthesis and crystal structure of a new 2D layered cadmium(II) coordination polymer: [Cd(bimc)_2_]n (bimc = 1H-Benzimidazole-5-carboxylate). Chin. J. Struct. Chem..

[122] Zhang J.Z., Cao W.R., Pan J.X., Chen Q.W. (2007). A novel two-dimensional square grid cobalt complex: Synthesis, structure, luminescent and magnetic properties. Inorg. Chem. Commun..

[123] Guo Z.G., Li X.J., Gao S.Y., Li Y.F., Cao R. (2007). A new three-dimensional supramolecular network, [Cd(Hbic)_2_(H_2_O)]·(4,4′-bpy) ·H_2_O (H_2_bic=1-H-benzimidazole-5carboxylic acid; 4,4-bpy=4,4′-bipyridine): Synthesis, crystal structure and luminescence property. J. Mol. Struct..

[124] Wei Y.Q., Yu Y.F., Wu K.C. (2008). Highly stable five-coordinated Mn(II) polymer [Mn(Hbidc)]_n_ (Hbidc=1*H*-Benzimidazole-5,6-dicarboxylate): Crystal structure, antiferromegnetic property, and strong long-lived luminescence. Cryst. Growth Des..

[125] Yao Y.L., Che Y.X., Zheng J.M. (2008). The coordination chemistry of benzimidazole-5,6-dicarboxylic acid with Mn(II), Ni(II), and Ln(III) complexes (Ln = Tb, Ho, Er, Lu). Cryst. Growth Des..

[126] Martinez-Lorente M.-A., Tuchagues J.-P., Petrouleas V., Savariault J.-M., Poinsot R., Drillon M. (1991). Bis(4-imidazoleacetato)iron.bis(methanol): a 2D antiferromagnetic iron(II) system exhibiting 3D long-range ordering with a net magnetic moment at 15 K. Inorg. Chem..

[127] Sun W.Y., Zhang Y.A., Okamura T., Ye N., Ueyama N. (2000). Synthesis and crystal structure of a new two-dimensional coordination polymer, {[Co^II^(imbz)_2_]·H_2_O}_n_ [imbz^-^ = 4-(Imidazol-1-ylmethyl)benzoate anion]. Chem. Lett..

[128] Zhao X.X., Ma J.P., Dong Y.B., Huang R.Q., Lai T.S. (2007). Construction of metal−organic frameworks (M = Cd(II), Co(II), Zn(II), and Cu(II)) based on semirigid oxadiazole bridging ligands by solution and hydrothermal reactions. Cryst. Growth Des..

[129] Ding D.G., Xu H., Fan Y.T., Hou H.W. (2008). Anion-dependent assemblies of two unprecedented copper(II) polymers with four-fold screw axes and trapped sodium chains. Inorg. Chem. Commun..

[130] Wang D.E., Wang F., Meng X.G., Ding Y., Wen L.L., Li D.F., Lan S.M. (2008). Syntheses, crystal structures and luminescent properties of three inorganic-organic hybrid frameworks constructed from 4,5-imidazoledicarboxylate. Z. Anorg. Allg. Chem..

[131] Gao S., Huo L.H., Zhao H., Liu J.W. (2005). Poly[aquamanganese(II)-*μ*_3_-1H-imidazole-4,5-dicarboxylato]. Acta Crystallogr..

[132] Lu W.G., Gu J.Z., Jiang L., Tan M.Y., Lu T.B. (2008). Achiral and chiral coordination polymers containing helical chains: the chirality transfer between helical chains. Cryst. Growth Des..

[133] Lu J.Y., Ge Z.H. (2005). Synthesis and structures of two new metal–organic polymers containing imidazoldicarboxylate ligands for hydrogen bonding networks, one with a covalent pleated sheet conformation. Inorg. Chim. Acta.

[134] Chen L., Bu X.H. (2006). Histidine-controlled two-dimensional assembly of zinc phosphite four-ring units. Chem. Mater..

[135] Shi W., Chen X.Y., Xu N., Song H.B., Zhao B., Cheng P., Liao D.Z., Yan S.P. (2006). Synthesis, crystal structures, and magnetic properties of 2D manganese(II) and 1D gadolinium(III) coordination polymers with 1*H*-1,2,3-triazole-4,5-dicarboxylic acid. Eur. J. Inorg. Chem..

[136] Yue Y.F., Liang J., Gao E.Q., Fang C.J., Yan Z.G., Yan C.H. (2008). Supramolecular engineering of a 2D Kagomé lattice: Synthesis, structures, and magnetic properties. Inorg. Chem..

[137] Qin J., Ma J.P., Liu L.L., Huang R.Q., Dong Y.B. (2009). A novel two-dimensional framework based on unprecedented cadmium(II) chains. Acta Crystallogr..

[138] Zhang X.F., Gao S., Huo L.H., Zhao H., Zhao J.G. (2006). Synthesis and crystal structure of 2D coordination polymer [Mn(HIDC)(H_2_O)]_n_ constructed by 1H-imidazole-4,5-dicarboxylate ligand. Chin. J. Inorg. Chem..

[139] Zhang X.F., Gao S., Huo L.H., Zhao H. (2007). Poly[[aquazinc(II)]-*μ*_3_-imidazole-4,5-dicarboxylato]. Acta Crystallogr..

[140] Zhang X.F., Gao S., Huo L.H., Zhao H. (2007). A two-dimensional cadmium(II) coordination polymer with unusual 4.8^2^ topology: poly[aqua(*μ*_3_-1H-imidazole-4,5-dicarboxylato)cadmium(II)]. Acta Crystallogr..

[141] Wang Y.T., Tang G.M., Wu Y., Qin X.Y., Qin D.W. (2007). Metal-controlled assembly tuning the topology and dimensionality of coordination polymers of Ag(I), Cd(II) and Zn(II) with the flexible 2-(1H-imidazole-1-yl)acetic acid (Hima). J. Mol. Struct..

[142] Wu C.D., Ayyappan P., Evans O.R., Lin W.B. (2007). Synthesis and x-ray structures of cadmium coordination polymers based on new pyridine−carboxylate and imidazole−carboxylate linkers. Cryst. Growth Des..

[143] Hu T.L., Du W.P., Hu B.W., Li J.R., Bu X.H., Cao R. (2008). Novel Ag(I) complexes with azole heterocycle ligands bearing acetic acid group: synthesis, characterization and crystal structures. CrystEngComm..

[144] Li X.Z., Qu Z.R. (2008). Poly[aqua[*μ*_3_-5-(2-carboxylatophenyl)-1H-tetrazolato]zinc(II)]. Acta Crystallogr..

[145] Li X.Z., Wu B.Z., Qu Z.R. (2008). Poly[diaqua-1*κ*^2^O-bis[*μ*_3_-2-(1H-tetrazol-5-yl)benzoate-(2)]dicadmium(II)]. Acta Crystallogr..

[146] Frisch M., Cahill C.L. (2005). Syntheses, structures and fluorescent properties of two novel coordination polymers in the U–Cu–H_3_pdc system. Dalton Trans..

[147] Zou W.Q., Wang M.S., Li Y., Wu A.Q., Zheng F.K., Chen Q.Y., Guo G.C., Huang J.S. (2007). Unprecedented (3,10)-connected 2-D metal-organic framework constructed from octanuclear cobalt(II) clusters and a new bifunctional ligand. Inorg. Chem..

[148] Yao M.X., Zeng M.H., Zou H.H., Zhou Y.L., Liang H. (2008). A unique 2D framework containing linear trimeric cobalt(II) of mixed *T*_d_–*O*_h_–*T*_d_ geometries linked by two different single-carboxylate-aromatic amine ligands: structure and magnetic properties. Dalton Trans..

[149] Lin J.D., Cheng J.W., Du S.W. (2008). Five d^10^ 3D metal−organic frameworks constructed from aromatic polycarboxylate acids and flexible imidazole-based ligands. Cryst. Growth Des..

[150] Liu Y.H., Wu H.C., Lin H.M., Hou W.H., Lu K.L. (2003). Crystal engineering toward intersecting channels in a interpenetrated diamondoid network based on a net-to-net H-bonding interaction. Chem. Commun..

[151] Zou R.Q., Zhong R.Q., Jiang L., Yamada Y., Kuriyama N., Xu Q. (2006). Tuning the formation of cadmium(II) urocanate frameworks by control of reaction conditions: crystal structure, properties, and theoretical investigation. Chem. Asian J..

[152] Zou R.Q., Yamada Y., Xu Q. (2006). Strong fluorescent emission of a new fourfold-interpenetrated diamondoid metal-organic framework of zinc(II) urocanate with one-dimensional open channels. Microporous Mesoporous Mater..

[153] Pan L., Huang X.Y., Li J. (2001). Assembly of new coordination frameworks in a pH-controlled medium: Syntheses, structures, and properties of ^3^_∞_[Cd(Hpdc)(H_2_O)] and ^3^_∞_[Cd_3_(pdc)_2_(H_2_O)_2_]. J. Solid State Chem..

[154] Li J.T., Tao J., Huang R.B., Zhang L.S. (2005). Poly[*μ*_4_-5-(3-carboxylatophenyl)-1H-tetrazolato-zinc(II)]. Acta Crystallogr..

[155] Du M., Zhang Z.H., Tang L.F., Wang X.G., Zhao X.J., Batten S.R. (2007). Molecular tectonics of metal-organic frameworks (MOFs): A rational design strategy for unusual mixed-connected network topologies. Chem. Eur. J..

[156] Zhang X.F., Gao S., Huo L.H., Zhao H. (2006). A three-dimensional porous cadmium(II) coordination polymer: poly[[(pyridine-*κ*N)cadmium(II)]-*μ*_3_-imidazole-4,5-dicarboxylato-*κ*^6^N,O:N′,O′:O′,O′′]. Acta Crystallogr..

[157] Zhang W.X., Xue W., Lin J.B., Zheng Y.Z., Chen X.M. (2008). 3D geometrically frustrated magnets assembled by transition metal ion and 1,2,3-triazole-4,5-dicarboxylate as triangular nodes. CrystEngComm..

[158] Wang Y., Shen Y.Z. (2008). A novel three-dimensional heterometallic coordination polymer: poly[[hexaaquabis[μ3-3,5-dicarboxylatopyrazolato-κ5O3,N2:N1,O5:O5′](μ2-oxalato-κ4O1,O2:O1′, O2′) copper(II)dierbium(III)] trihydrate]. Acta Crystallogr.

[159] Wang Y.L., Yuan D.Q., Bi W.H., Li X., Li X.J., Li F., Cao R. (2005). Syntheses and characterizations of two 3D cobalt−organic frameworks from 2D honeycomb building blocks. Cryst. Growth Des..

[160] King P., Clérac R., Anson C.E., Coulon C., Powell A.K. (2003). Antiferromagnetic three-dimensional order induced by carboxylate bridges in a two-dimensional network of [Cu_3_(dcp)_2_(H_2_O)_4_] trimers. Inorg.Chem..

[161] Liu W.L., Ye L.H., Liu X.F., Yuan L.M., Lu X.L., Jiang J.X. (2008). Rapid synthesis of a novel cadmium imidazole-4,5-dicarboxylate metal-organic framework under microwave-assisted solvothermal condition. Inorg. Chem. Commun..

[162] Sang R.L., Xu L. (2008). Unprecedented helix-based microporous metal–organic frameworks constructed from a single ligand. Chem.Commun..

[163] Zhao J., Long L.S., Huang R.B., Zheng L.S. (2008). A lanthanide-based metal–organic framework with a dynamic porous property. Dalton Trans..

[164] Li C.J., Hu S., Li W., Lam C.K., Zheng Y.Z., Tong M.L. (2006). Rational design and control of the dimensions of channels in three-dimensional, porous metal-organic frameworks constructed with predesigned hexagonal layers and pillars. Eur. J. Inorg. Chem..

[165] Lu W.G., Jiang L., Feng X.L., Lu T.B. (2006). Three 3D coordination polymers constructed by Cd(II) and Zn(II) with imidazole-4,5-dicarboxylate and 4,4′-bipyridyl building blocks. Cryst. Growth Des..

[166] Zhang M.B., Chen Y.M., Zheng S.T., Yang G.Y. (2006). A 3D manganese coordination polymer [Mn_3_(IMDC)_2_(H_2_O)_4_] constructed from [Mn_2_(IMDC)_2_(H_2_O)_2_] layers and [Mn(H_2_O)_2_] pillars (IMDC = 4,5-imidazoledicarboxylate). Eur. J. Inorg. Chem..

[167] Yao Y.L., Che Y.X., Zheng J.M. (2008). A new eight-connected CsCl-type net using bicadmium cores as nodes. Inorg. Chem. Commun..

[168] Zhong R.Q., Zou R.Q., Xu Q. (2007). Microporous metal-organic framework zinc(II) imidazole- 4,5-dicarboxylate: Four-fold helical structure and strong fluorescent emission. Microporous Mesoporous Mater..

[169] Lu W.G., Jiang L., Feng X.L., Lu T.B. (2008). four 3d porous metal−organic frameworks with various layered and pillared motifs. Cryst. Growth Des..

[170] Cahill C.L., de Lill D.T., Frisch M. (2007). Homo- and heterometallic coordination polymers from the f elements. CrystEngComm.

[171] Gu J.Z., Lu W.G., Jiang L., Zhou H.C., Lu T.B. (2007). 3D porous metal-organic framework exhibiting selective adsorption of water over organic solvents. Inorg. Chem..

[172] Wang S., Zhang L.R., Li G.H, Huo Q.S., Liu Y.L. (2008). Assembly of two 3-D metal–organic frameworks from Cd(II) and 4,5-imidazoledicarboxylic acid or 2-ethyl-4,5-imidazole-dicarboxylic acid. CrystEngComm.

[173] Pan L., Huang X.Y., Li J., Wu Y.G., Zheng N.W. (2000). Novel single- and double-layer and three-dimensional structures of rare-earth metal coordination polymers: The effect of lanthanide contraction and acidity control in crystal structure formation. Angew. Chem., Int. Ed..

[174] Sun Y.Q., Yang G.Y. (2007). Organic–inorganic hybrid materials constructed from inorganic lanthanide sulfate skeletons and organic 4,5-imidazoledicarboxylic acid. Dalton Trans..

[175] Sun Y.Q., Zhang J., Yang G.Y. (2006). A series of luminescent lanthanide–cadmium–organic frameworks with helical channels and tubes. Chem. Commun..

